# Identification of inadequate responders to advanced therapy among commercially-insured adult patients with Crohn’s disease and ulcerative colitis in the United States

**DOI:** 10.1186/s12876-023-02675-w

**Published:** 2023-03-09

**Authors:** Theresa Hunter Gibble, April N. Naegeli, Michael Grabner, Keith Isenberg, Mingyang Shan, Chia-Chen Teng, Jeffrey R. Curtis

**Affiliations:** 1 Eli Lilly and Company, Indianapolis, IN USA; 2 HealthCore, Inc., Wilmington, DE USA; 3 Anthem, Inc., Indianapolis, IN USA; 4 University of Alabama at Birmingham, Birmingham, AL USA

**Keywords:** Advanced therapy, Biologics, Claims-based algorithm, Crohn’s disease, HealthCore Integrated Research Database^®^, Inadequate response, Inflammatory bowel disease, Tumor necrosis factor inhibitors/TNFi, Ulcerative colitis

## Abstract

**Background:**

The purpose of this analysis was to assess the frequency of inadequate response over 1 year from advanced therapy initiation among patients with Crohn’s disease (CD) or ulcerative colitis (UC) in the United States using a claims-based algorithm. Factors associated with inadequate response were also analyzed.

**Methods:**

This study utilized claims data of adult patients from the HealthCore Integrated Research Database (HIRD^®^) from January 01, 2016 to August 31, 2019. Advanced therapies used in this study were tumor necrosis factor inhibitors (TNFi) and non-TNFi biologics. Inadequate response to an advanced therapy was identified using a claims-based algorithm. The inadequate response criteria included adherence, switching to/added a new treatment, addition of a new conventional synthetic immunomodulator or conventional disease-modifying drugs, increase in dose/frequency of advanced therapy initiation, and use of a new pain medication, or surgery. Factors influencing inadequate responders were assessed using multivariable logistic regression.

**Results:**

A total of 2437 patients with CD and 1692 patients with UC were included in this analysis. In patients with CD (mean age: 41 years; female: 53%), 81% had initiated TNFi, and 62% had inadequate response. In patients with UC (mean age: 42 years; female: 48%), 78% had initiated a TNFi, and 63% had an inadequate response. In both patients with CD and UC, inadequate response was associated with low adherence (CD: 41%; UC: 42%). Inadequate responders were more likely to be prescribed a TNFi (for CD: odds ratio [OR] = 1.94; *p* < 0.001; for UC: OR = 2.76; *p* < 0.0001).

**Conclusion:**

More than 60% of patients with CD or UC had an inadequate response to their index advanced therapy within 1 year after initiation, mostly driven by low adherence. This modified claims-based algorithm for CD and UC appears useful to classify inadequate responders in health plan claims data.

**Supplementary Information:**

The online version contains supplementary material available at 10.1186/s12876-023-02675-w.

## Background

Crohn’s disease (CD) and ulcerative colitis (UC) are chronic progressive inflammatory diseases of the gastrointestinal (GI) tract, collectively referred to as inflammatory bowel disease (IBD). While CD can affect the entire GI tract, UC mostly remains limited to the colonic mucosa [1]. IBD is estimated to affect more than 6.8 million individuals globally [1]. In the United States (US), 3.1 million adults are known to be affected (2015 estimates); age-standardized prevalence in the US was estimated to be 464.5 patients per 100,000 population [1, 2]. IBD imposes significant health and economic burden on communities worldwide, and substantially impacts patients’ quality of life [1].

Conventional treatments for both CD and UC include corticosteroids, aminoacylates, antibiotics, and immunomodulatory drugs [3]. Advanced therapies, such as tumor necrosis factor inhibitors (TNFi), interleukin (IL) 23 p40 inhibitors, integrin inhibitors or Janus kinase inhibitors (only for UC) are usually reserved for patients with moderate-to-severe IBD, who do not adequately respond to conventional therapies [3, 4]. Despite the availability of newer therapies, TNFi drugs remain the first-line treatment of moderate-to-severe IBD. These drugs have been shown to be well-tolerated and effective for inducing and maintaining the remission of CD and UC [5, 6]. However, about 10–40% of patients with IBD are primary non-responders [7] and up to 50% of patients experience secondary loss of response after 12 months on therapy [8]. Inadequate response to advanced therapies indicates a need for newer therapies to improve the management in patients with CD or UC. Therefore, investigating treatment effectiveness in patients with CD and UC would help clinicians make more informed treatment decisions and contribute to value-based reimbursement models of care.

In the recent past, various algorithms have been used to assist researchers and clinicians in identifying patients with CD or UC, treatment patterns, treatment safety and healthcare resource utilization from insurance claims database studies [9–13]. While effectiveness and safety outcomes associated with effective treatment in CD and UC have been studied using these types of data [14, 15], methods of evaluating the inadequate response to medications in patients with CD and UC from claims databases are limited. The present study investigated the frequency of, and factors associated with, inadequate response over 1 year after advanced therapy initiation in adult patients from the US. Inadequate responses were identified by using a claims-based algorithm originally developed by Curtis et al. [16], validated for rheumatoid arthritis and adapted for use in IBD.

## Methods

### Data source and population

This was a retrospective claim-based cohort study that utilized longitudinal claims data from the HealthCore Integrated Research Database^®^ (HIRD^®^) from January 1, 2016 to August 31, 2019. The HIRD^®^ contains data from January 2006 on patient enrollment, inpatient and outpatient medical care, prescription, and health care utilization. It is a large longitudinal medical and pharmacy claims database of health plan members comprising all regions of the US.

The data were accessed and used in full compliance with the relevant provisions of the Health Insurance Portability and Accountability Act. The study was conducted under the research provisions of Privacy Rule 45 CFR 164.514(e). Researchers’ access to claims data was limited to data stripped of identifiers to ensure confidentiality. An Institutional Review Board did not review the study since only this limited data set was accessed. This study was conducted in accordance with the ethical principles that have their origin in the Declaration of Helsinki and that are consistent with Good Pharmacoepidemiology Practices as well as legal and regulatory requirements.

Adult patients aged ≥ 18 years with CD (International Classification of Diseases, 10th Revision, Clinical Modification [ICD-10-CM] diagnosis codes: K50.x) or UC (ICD-10-CM diagnosis codes: K51.x) who initiated an advanced therapy during the index period of July 1, 2016 through August 31, 2018 were included in the study. Index date was defined as the first observed occurrence of a claim (medical or pharmacy) for any eligible advanced therapy during the index period. For patients who started more than one therapy, only the earliest one observed was used. Included patients were enrolled in commercial, Medicare Advantage, or Medicare Supplemental plus Part D insurance plans for ≥ 6 months before the index date (pre-index period) and ≥ 12 months after index date (follow-up period). Eligible patients were required to have ≥ 2 medical claims for CD or UC from a provider of any specialty at least seven days apart during the study period, of which ≥ 1 claim occurred during the pre-index period.

In this study, advanced therapies for CD included TNFi (adalimumab, certolizumab, infliximab) and non-TNFi (natalizumab, ustekinumab, vedolizumab). For UC, advanced therapies included TNFi (adalimumab, golimumab, infliximab), non-TNFi (vedolizumab; ustekinumab as a potential switcher but not index drug), and other therapies (tofacitinib). Conventional therapies included 5-aminosalicylic acid derivatives (mesalazine and sulfasalazine) and immunosuppressants (azathioprine, methotrexate, mycophenolate, cyclosporine, tacrolimus, 6-mercaptopurine).

Patients were excluded if they had claims for ≥ 1 advanced therapy during the 6-month pre-index period to identify new initiators of advanced therapy. Patients who had evidence for other autoimmune diseases including psoriasis, lupus, ankylosing spondylitis, psoriatic arthritis, or rheumatoid arthritis (defined as ≥ 2 claims on different dates for the same disease) were also excluded in order to avoid misclassification of the estimated response rate (e.g., related to non-adherence) due to multiple indications.

### Criteria of inadequate response

The algorithm to identify inadequate response to index advanced therapy was derived from a claims-based algorithm originally developed by Curtis et al. [16] and validated for rheumatoid arthritis. The first claim for advanced therapy is set as index date. Some modifications were made to the algorithm for UC and CD. The absence of all criteria listed in Table 1 denoted adequate response (stable disease); presence of one or more of them denoted inadequate response. For example, low index therapy adherence reflects inadequate response. All criteria were calculated based on the 1-year follow-up period for each patient. Details of the algorithm used are presented in Additional file 1: Table S1. In brief, the parameters of the algorithm included low adherence (defined as proportion of days covered [PDC] < 80%), switched/added new advanced therapy/new biologic, added a new conventional therapy, increased dose/frequency of advanced therapy/biologics, addition or dose increase of oral glucocorticoids, used a new pain medication, or had surgery for UC or CD.

**Table 1:** Inadequate response criteria evaluated over 1-year follow-up for both Crohn’s disease and ulcerative colitis

**Criteria based on the reference algorithm** [16]
Low adherence to index advanced therapy (defined as proportion of days covered [PDC] < 80%)
Switch/add non-index advanced therapy
Add new conventional therapy (methotrexate, sulfasalazine, and others)
Dose or frequency increase of index advanced therapy (> 20% higher than the index dose)
Addition or dose increase of oral glucocorticoid
**Additional criteria for this study**
Use of pain medication class^a^ not observed at pre-index period
Use of surgery (Current Procedural Terminology codes for surgery are presented in Additional file 1: Table S2)

^a^Opioids, nonsteroidal anti-inflammatory drugs, non-narcotic analgesics, neuromodulators (anti-depressants, anticonvulsants, muscle relaxants)

### Variables measured

Patient characteristics (age, sex, geographic region, Quan-Charlson Comorbidity Index [QCI] score, and specific comorbidities), provider specialty, and prior/historical treatments were assessed during pre-index period or on the index date. Prior “historic exposure” was defined as any claim from start of the patient’s continuous plan enrollment time up until the beginning of the 6-month pre-index period. Inadequate response to advanced therapies was recorded during 1-year follow-up and compared between patients receiving TNFi and non-TNFi.

### Statistical analysis

Descriptive statistics including mean, standard deviation (SD), median, and absolute/relative frequencies for continuous and categorical data, respectively, were reported. Patient characteristics were statistically compared between responders and inadequate responders using Chi-square tests or Fisher’s exact tests for dichotomous variables, and t-tests or Wilcoxon tests for continuous variables. Characteristics during pre-index or on index date associated with inadequate response to the index advanced therapy were identified by a multivariable logistic regression model using a stepwise selection with entrance and exit *p* value cut-offs of 0.15. Index drug class, age, and gender were included in the model *a-priori*. Variables with prevalence rate < 1% in any group (responder or inadequate responder) were excluded from model selection. Goodness-of-fit statistics including C-statistic (higher score preferred) and Hosmer–Lemeshow test (*p* value > 0.05 preferred) were reported for each logistic regression. An alpha level of 0.05 was considered as statistically significant without any adjustments made for multiple comparisons. Sample selection and creation of analytic variables were performed using the Instant Health Data platform (Panalgo, Boston, MA). Statistical analyses were performed using SAS version 9.3 (SAS Institute Inc., Cary, NC, USA).

## Results

### Patient characteristics

Of the 140,435 patients who initiated any advanced therapy identified in the HIRD^®^, 2437 patients with CD and 1692 with UC were included in this analysis. Patient selection details are presented in Additional file 1: Fig. S1.

Mean (SD) age of patients with CD was 41 (14.7) years. Approximately 53% of patients with CD were female and the majority (60%) was covered under preferred provider organization (PPO) health plans. During the pre-index period, mean QCI score was 0.40, and infection was the most common (~ 38%) among all comorbidities. In patients with CD who initiated a TNFi as their index therapy, 53% received adalimumab. Over the 1-year follow-up period, 62% of patients with CD had an inadequate response to their index biologic therapies. Compared with responders, the proportion of female patients (49% vs. 56%) was higher among inadequate responders. QCI score and mental health issues were higher in inadequate responders compared with responders (*p* < 0.05). Use of any conventional therapy was higher in responders except methotrexate versus inadequate responders (50% vs 42%, respectively). Overall, there was no difference in the use of TNFi and non-TNFi therapies (vedolizumab and ustekinumab) as the starting medication on index date. Adalimumab use was higher in responder group, and certolizumab and infliximab were higher among inadequate responder (Table 2).

**Table 2:** Baseline demographic and clinical characteristics of Crohn’s disease patients at biologic index date

	Patients with CD N = 2437	Responders n = 921	Inadequate responders n = 1516	*p* value
Age, years; mean (SD)	40.8 (14.7)	41.0 (14.5)	40.7 (14.8)	0.5088
Gender, n (%)				
Female	1299 (53.3%)	447 (48.5%)	852 (56.2%)	**0.0002**
Health plan type, n (%)				
HMO	441 (18.1%)	171 (18.6%)	270 (17.8%)	0.6380
PPO	1441 (59.1%)	561 (60.9%)	880 (58.1%)	0.1631
CDHP	555 (22.8%)	189 (20.5%)	366 (24.1%)	**0.0387**
Geographic region^a^; n (%)				
Northeast	352 (14.4%)	132 (14.3%)	220 (14.5%)	0.9027
Midwest	626 (25.7%)	253 (27.5%)	373 (24.6%)	0.1164
South	969 (39.8%)	348 (37.8%)	621 (41.0%)	0.1201
West	369 (15.1%)	147 (16.0%)	222 (14.6%)	0.3791
Index year, n (%)				
2016	621 (25.5%)	223 (24.2%)	398 (26.3%)	0.2624
2017	1130 (46.4%)	434 (47.1%)	696 (45.9%)	0.5606
2018	686 (28.2%)	264 (28.7%)	422 (27.8%)	0.6594
Quan-Charlson comorbidity index, mean (SD)	0.40 (0.90)	0.36 (0.85)	0.42 (0.92)	**0.0488**
Comorbid conditions, n (%)				
Anemia	585 (24.0%)	208 (22.6%)	377 (24.9%)	0.2006
Dyslipidemia	632 (25.9%)	224 (24.3%)	408 (26.9%)	0.1570
Hypertension	518 (21.3%)	195 (21.2%)	323 (21.3%)	0.9378
Infections	936 (38.4%)	332 (36.1%)	604 (39.8%)	0.0619
Mental health issues^b^	740 (30.4%)	249 (27.0%)	491 (32.4%)	**0.0053**
Anxiety or depression^c^	528 (21.7%)	172 (18.7%)	356 (23.5%)	**0.0052**
Other mental health issue (excluding anxiety/depression)	358 (14.7%)	116 (12.6%)	242 (16.0%)	**0.0228**
Any conventional therapy^d^, n (%)	1085 (44.5%)	456 (49.5%)	629 (41.5%)	**0.0001**
Historical use of TNFi, n (%)	152 (6.2%)	37 (4.0%)	115 (7.6%)	**0.0004**
Index advance therapy, n (%)				
TNFi				
Adalimumab	1279 (52.5%)	500 (54.3%)	779 (51.4%)	0.1640
Certolizumab	61 (2.5%)	14 (1.5%)	47 (3.1%)	**0.0155**
Infliximab	625 (25.7%)	224 (24.3%)	401 (26.5%)	0.2430
Non-TNFi				
Ustekinumab	198 (8.1%)	65 (7.1%)	133 (8.8%)	0.1328
Vedolizumab	270 (11.1%)	117 (12.7%)	153 (10.1%)	**0.0464**

*p* < 0.05 was considered as statistically significant

CD Crohn's disease, CDHP Consumer-driven health plan, *HMO* Health management organization, *ICD-10-CM* International Classification of Diseases, 10th Revision, Clinical Modification, *PPO* Preferred provider organization, *TNFi* Tumour necrosis factor alpha inhibitor

^a^Based on US census regions; remainder is Other/Unknown

^b^Includes ICD-10-CM diagnosis codes F01 to F69 (mental disorders due to known physiological conditions, psychoactive substance use, schizophrenia, schizotypal, delusional, and other non-mood psychotic disorders, mood [affective] disorders, anxiety, dissociative, stress-related, somatoform and other nonpsychotic mental disorders, behavioural syndromes associated with physiological, disturbances and physical factors, disorders of adult personality and behaviour)

^c^ICD-10-CM diagnosis codes (anxiety: F41-F48; depression: F32-F33)

^d^Conventional therapy includes 5-aminosalicylic acid derivatives (mesalazine and sulfasalazine) and immunosuppressants (azathioprine, methotrexate, mycophenolate, cyclosporine, tacrolimus, 6-mercaptopurine) were considered conventional therapy

Mean (SD) age of patients with UC was 42 (14.9) years. Approximately 48% of patients with UC were female and the majority (62%) was covered under PPO health plans. During the pre-index period, infection was the most common (~ 37%) among all comorbidities. Approximately 78% of patients with UC initiated a TNFi as index therapy, of which 48% received adalimumab. Over the 1-year follow-up period, 63% of patients with UC had an inadequate response to their index advanced therapies. Use of any conventional therapies such as 5-aminosalicylic acid derivatives (mesalazine and sulfasalazine) and immunosuppressants (azathioprine, methotrexate, mycophenolate, cyclosporine, tacrolimus, 6-mercaptopurine) during the pre-index period was lower among inadequate responders (80%) compared with responders (85%). Similarly, the use of non-TNFi therapies (vedolizumab) as the starting medication was lower among inadequate responders (16%) versus responders (33%, *p* < 0.0001). In contrast, use of TNFi therapies as the index medication was higher among inadequate responders (84%) compared with adequate responders (67%) (Table 3).

**Table 3:** Baseline demographic and clinical characteristics of ulcerative colitis patients at biologic index date

	Patients with UC N = 1692	Responders n = 619	Inadequate responders n = 1073	*p* value
Age, years; mean (SD)	42.3 (14.9)	42.3 (14.6)	42.3 (15.1)	0.9804
Gender, n (%)				
Female	806 (47.6%)	300 (48.5%)	506 (47.2%)	0.6039
Health plan type, n (%)				
HMO	270 (16.0%)	92 (14.9%)	178 (16.6%)	0.3503
PPO	1046 (61.8%)	391 (63.2%)	655 (61.0%)	0.3867
CDHP	376 (22.2%)	136 (22.0%)	240 (22.4%)	0.8502
Geographic region^a^, n (%)				
Northeast	276 (16.3%)	98 (15.8%)	178 (16.6%)	0.6848
Midwest	378 (22.3%)	139 (22.5%)	239 (22.3%)	0.9312
South	616 (36.4%)	231 (37.3%)	385 (35.9%)	0.5539
West	335 (19.8%)	123 (19.9%)	212 (19.8%)	0.9552
Index year, n (%)				
2016	384 (22.7%)	136 (22.0%)	248 (23.1%)	0.5891
2017	764 (45.2%)	279 (45.1%)	485 (45.2%)	0.9595
2018	544 (32.2%)	204 (33.0%)	340 (31.7%)	0.5902
Quan-Charlson comorbidity index, mean (SD)	0.40 (1.01)	0.32 (0.84)	0.44 (1.11)	0.1145
Comorbid conditions, n (%)				
Anemia	417 (24.7%)	157 (25.4%)	260 (24.2%)	0.6026
Dyslipidemia	456 (27.0%)	165 (26.7%)	291 (27.1%)	0.8357
Hypertension	332 (19.6%)	112 (18.1%)	220 (20.5%)	0.2293
Infections	632 (37.4%)	225 (36.4%)	407 (37.9%)	0.5170
Mental health issues^b^	408 (24.1%)	139 (22.5%)	269 (25.1%)	0.2260
Anxiety or depression^c^	323 (19.1%)	108 (17.5%)	215 (20.0%)	0.1917
Other mental health issue (excluding anxiety/depression)	157 (9.3%)	48 (7.8%)	109 (10.2%)	0.1007
Any conventional therapy^d^, n (%)	1386 (81.9%)	524 (84.7%)	862 (80.3%)	**0.0263**
Historical use of TNFi, n (%)	46 (2.7%)	14 (2.3%)	32 (3.0%)	0.3800
Index advanced therapy, n (%)				
TNFi				
Adalimumab	806 (47.6%)	260 (42.0%)	546 (50.9%)	**0.0004**
Golimumab	57 (3.4%)	19 (3.1%)	38 (3.5%)	0.6042
Infliximab	450 (26.6%)	134 (21.7%)	316 (29.5%)	**0.0005**
Non-TNFi				
Vedolizumab	376 (22.2%)	205 (33.1%)	171 (15.9%)	< **0.0001**

*p*<0.05 was considered as statistically significant

*CDHP* Consumer-driven health plan, *HMO* Health management organization, *ICD-10-CM* International Classification of Diseases, 10th Revision, Clinical Modification,  *PPO* Preferred provider organization, *TNFi* Tumour necrosis factor alpha inhibitor, *UC* Ulcerative colitis

^a^Based on US census regions; remainder is other/unknown

^b^Includes ICD-10-CM diagnosis codes F01 to F69 (mental disorders due to known physiological conditions, psychoactive substance use, schizophrenia, schizotypal, delusional, and other non-mood psychotic disorders, mood [affective] disorders, anxiety, dissociative, stress-related, somatoform and other nonpsychotic mental disorders, behavioural syndromes associated with physiological, disturbances and physical factors, disorders of adult personality and behaviour)

^c^ICD-10-CM diagnosis codes (anxiety: F41-F48; depression: F32-F33)

^d^Conventional therapy includes 5-aminosalicylic acid derivatives (mesalazine and sulfasalazine) and immunosuppressants (azathioprine, methotrexate, mycophenolate, cyclosporine, tacrolimus, 6-mercaptopurine) were considered conventional therapy

### Inadequate response algorithm details among patients with Crohn’s disease and ulcerative colitis

Among all patients with CD who were identified as having inadequate response to their index advanced therapy based on observed claims utilization patterns, 41% of patients had low adherence, 14% switched/added new advanced therapy, and 13% added new conventional therapies. Approximately 12% of patients had a dose/frequency increase of their index advanced therapy, 12% had an addition/dose increase of oral glucocorticoids, and 5% had surgery (Table 4).

**Table 4:** Inadequate responders during 12-month post-index period in patients with Crohn’s disease based on treatment class

	Patients with CD N = 2437	TNFi n = 1965	Non-TNFi n = 472
Inadequate response	1516 (62.2%)	1227 (62.4%)	289 (61.2%)
Criteria for inadequate response, n (%)			
Low adherence to index biologic (PDC < 80%)	1000 (41.0%)	813 (41.4%)	187 (39.6%)
Switch/add new biologic (on-label)	332 (13.6%)	284 (14.5%)	48 (10.2%)
Add new conventional therapy^a^	309 (12.7%)	262 (13.3%)	47 (10.0%)
Dose or frequency increase of index biologic	286 (11.7%)	253 (12.9%)	33 (7.0%)
Addition or dose increase of oral glucocorticoid	281 (11.5%)	210 (10.7%)	71 (15.0%)
Use of new pain medication	192 (7.9%)	159 (8.1%)	33 (7.0%)
Surgery	126 (5.2%)	96 (4.9%)	30 (6.4%)

Data presented as n (%)

*CD* Crohn’s disease,* PDC* Proportion of days covered, *TNFi* Tumor necrosis factor alpha inhibitors

^a^Conventional therapy includes methotrexate or sulfasalazine and others

Among patients with UC who had inadequate response, the majority had low adherence (42%), followed by 24% who switched/added new advanced therapy, and 18% added new conventional therapies. Approximately 13% had a dose/frequency increase of their index advanced therapies. Addition or increase in oral glucocorticoids dose was observed among 14% of patients, and 2% required surgery (Table 5).

**Table 5:** Inadequate responders during 12-month post index period in patients with ulcerative colitis based on treatment class

	Patients with UC N = 1692	TNFi n = 1313	Non-TNFi n = 376
Inadequate response, n (%)	1073 (63.4%)	900 (68.6%)	171 (45.5%)
Criteria for inadequate response, n (%)			
Low adherence to index biologic (PDC < 80%)	710 (42.0%)	608 (46.3%)	100 (26.6%)
Switch/add new biologic (on-label)	405 (23.9%)	355 (27.0%)	49 (13.0%)
Add new conventional therapy^a^	300 (17.7%)	253 (19.3%)	47 (12.5%)
Dose or frequency increase of index biologic	212 (12.5%)	NA^b^	NA^b^
Addition or dose increase of oral glucocorticoid	233 (13.8%)	190 (14.5%)	42 (11.2%)
Use of new pain medication	134 (7.9%)	109 (8.3%)	25 (6.7%)
Surgery	34 (2.0%)	24 (1.8%)	10 (2.7%)

Data presented as n (%). Inadequate response results were not reported for tofacitinib users due to small sample size

*TNFi* Tumor necrosis factor alpha inhibitors, *PDC* Proportion of days covered, *UC* Ulcerative colitis, *NA* Not available (small cell size masked to preserve patient privacy)

^a^Conventional therapy includes methotrexate, sulfasalazine and others

^b^Denotes n < 10, which was blinded for privacy

### Factors influencing inadequate response to advanced therapy

**Fig. 1:**
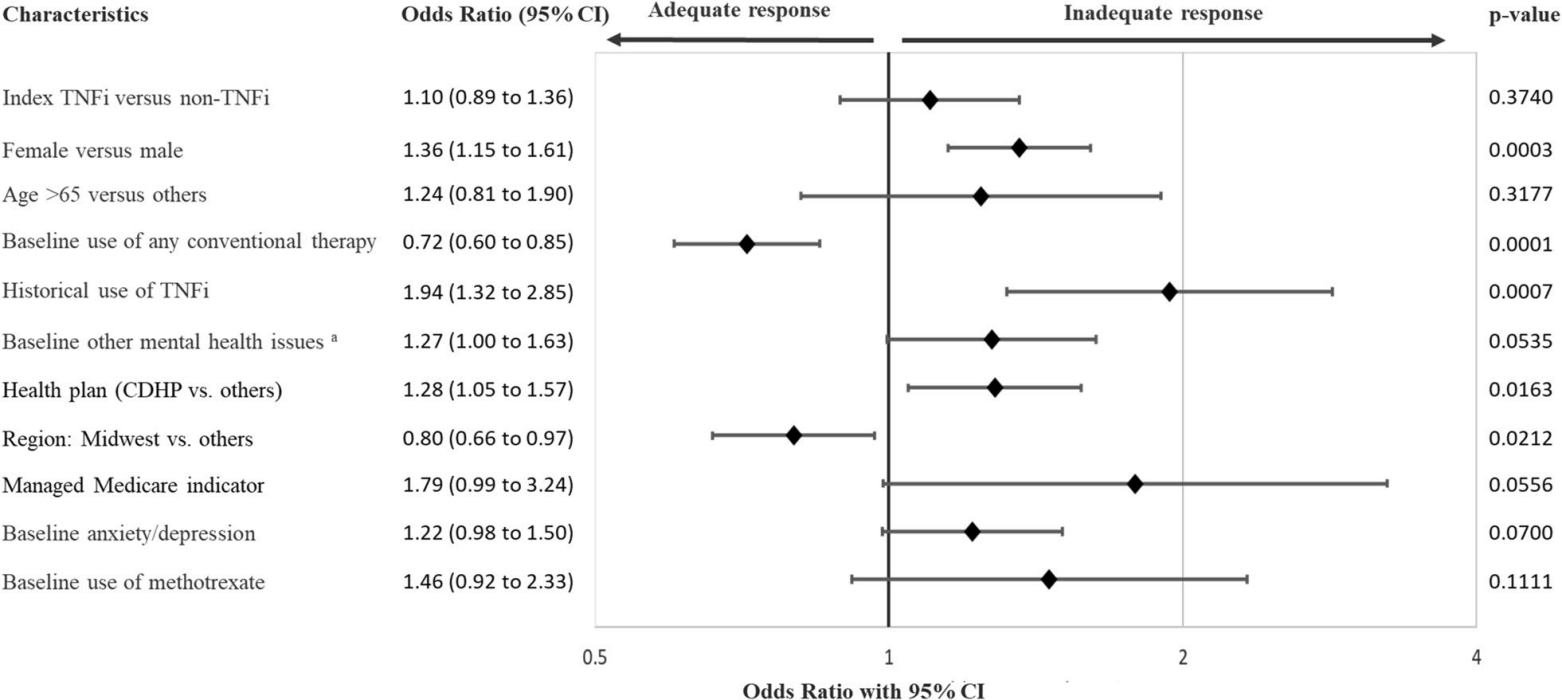
Association between baseline patient characteristics and inadequate response to advance therapies for Crohn’s disease. ^a^Excluding anxiety and depression. Historical use of TNFi: use of TNFi at any time prior to the 6-month pre-index period. Conventional therapy includes azathioprine, balsalazide, cyclosporine, hydroxychloroquine, leflunomide, mercaptopurine, mesalamine, methotrexate, minocycline, mycophenolate, olsalazine, sulfasalazine. *TNFi* Tumor necrosis factor alpha inhibitors

In patients with CD, inadequate responders were more likely to be female (odds ratio [OR] confidence interval [CI] = 1.36 [1.15 to 1.61]), have historical use of TNFi (OR [CI] = 1.94 [1.32 to 2.85]), and be on a consumer-driven health plan (OR [CI] = 1.28 [1.05 to 1.57]) (Fig. 1).

**Fig. 2:**
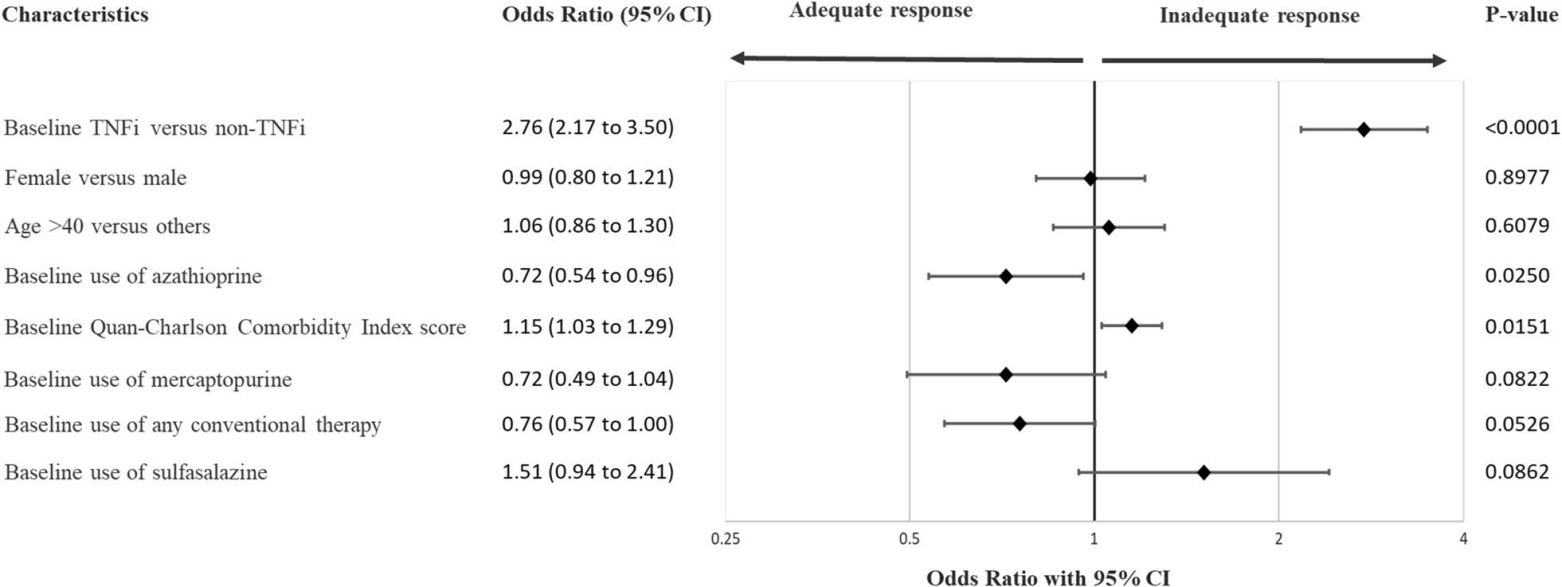
Association between baseline patient characteristics and inadequate response to advance therapies for ulcerative colitis. The 3 patients receiving tofacitinib were excluded from this model. Conventional therapy includes azathioprine, balsalazide, cyclosporine, hydroxychloroquine, leflunomide, mercaptopurine, mesalamine, methotrexate, minocycline, mycophenolate, olsalazine, sulfasalazine. *TNFi* Tumor necrosis factor alpha inhibitors

In patients with UC, inadequate responders were more likely to be prescribed a TNFi (OR [CI] = 2.76 [2.17 to 3.50]) and have a higher pre-index QCI Index (OR [CI] = 1.15 [1.03 to 1.29]) (Fig. 2).

Patients with CD and UC were more likely to be responder if they had pre-index use of any conventional therapy (OR [CI] = 0.72 [0.60 to 0.85]) or azathioprine (OR [CI] = 0.72 [0.54 to 0.96]), respectively (Figs. 1, 2).

## Discussion

In this retrospective claim-based study, an algorithm was developed to assess the extent of inadequate response to prescription treatments for the management of CD and UC. Adherence, increase in dose/frequency, and addition of new medications were the key indicators of inadequate response in this study. Results showed that most of the patients with CD or UC had initiated TNFi as first advanced therapy (~ 80%), of which ~ 50% started with adalimumab. However, a considerable proportion (> 60%) of patients did not adequately respond during the 1-year follow-up. The majority of both CD and UC inadequate responders were classified as such based upon low adherence to their index treatment (> 40%). The next most common reasons for inadequate response were switching to or adding new advanced therapy and increasing dose of existing therapies or glucocorticoids.

Results of this study are consistent with those observed in claims-based algorithm studies in rheumatoid arthritis [16] and in ankylosing spondylitis and psoriatic arthritis [17]. Approximately 70% of patients with rheumatoid arthritis, ankylosing spondylitis and psoriatic arthritis responded inadequately to advanced biologic treatments, where the most common reason for inadequate response was low adherence to the index medication [16, 17]. Adherence to medication is a major problem in the management of IBD as well which can lead to adverse clinical outcomes including an increase in disease activity, relapse, and loss of response to TNFi [18]. We used low adherence as a proxy for inadequate response to treatment as patients are unlikely to persist taking a medication that is ineffective or causes intolerance or adverse events, any of which may result in lack of effectiveness. Switching to advanced therapy, adding a new conventional therapy, increasing dose or frequency of medication including oral glucocorticoid, use of pain medication, are other prominent indicators to show that current therapies are not sufficient. In a proportion of patients, need for surgery may also indicate the insufficiency of current therapies. Inadequate response to TNFi typically leads to discontinuation of treatment. Other studies using real-world data showed that approximately half of patients with IBD receiving infliximab or adalimumab discontinued treatment during 1-year follow-up, and a substantial percentage were switched to a nonbiologic [10, 19]. Moreover, in patients initiating a new biologic therapy for IBD, the likelihood of providers tapering therapy within the first year due to adequate response, such that it would result in low measured adherence, is less.

Inadequate response to advanced therapy cannot be explained by biology alone; algorithm-based studies are required to identify factors influencing inadequate response. In our study, factors influencing inadequate response in patients with CD included being female, historical use of TNFi, and having a consumer-driven health plan. While in patients with UC, these factors included baseline use of TNFi and higher QCI score. Consistent with the observations from some clinical trials, such as the one comparing infliximab plus azathioprine combination therapy [20], patients taking any conventional therapy (CD) or azathioprine (UC) in the pre-index period in our study were more likely to be responders. A similar finding was also reported in a retrospective cohort study based on German claims data: Patients with UC who received conventional therapies at index showed less likelihood of experiencing inadequate response versus patients who were not on conventional therapy (Hazard ratio, 0.73; 95% CI 0.59–0.90); initial concomitant use of conventional therapies was associated with fewer dose augmentations [21].

To our knowledge, this is the first study to use a claims-based algorithm to serve as a proxy for the clinical effectiveness of treatments for CD and UC in the United States. Results of the study suggest that there is a large unmet need in the management of CD and UC for more effective therapies and disease management strategies which can sustain remission. In addition, this study showed that algorithms are a promising proxy to investigate inadequate response to advanced therapies utilizing claims databases, which could be useful for value-based contracts and other innovative reimbursement schemes relevant for health plans and other stakeholders with access to claims data.

Our study also has some limitations associated with claims-database analysis. Patients identified in this study may not be representative of the US population who receive health care through government organizations or who lack health insurance. The relationships described between baseline patient characteristics and responder status represent associations rather than causal chains; several important confounders, such as disease activity metrics (e.g., CDAI) or provider behaviors, are unavailable or incomplete in claims data. For patients who used multiple advanced therapies over time, we focused on the first of them to assess adequate response and did not examine subsequent treatments’ response rate. Prescription claims include medications dispensed by pharmacies, and do not necessarily reflect the actual consumption by patients. The possibility of coding errors associated with incorrect diagnoses cannot be completely avoided. Furthermore, the modified algorithm used in this analysis has yet to be validated against clinical UC or CD disease activity measures or biomarkers. The therapeutic landscape of CD and UC is constantly changing with the introduction of novel treatments whose indications also change over time; therefore, the use of therapies as assumed in this study may not fully reflect actual indications at each time point of the study period. Nevertheless, this study provides important insights into the ways in which biologic agents are currently used in clinical practice. Future studies are warranted for the validation, augmentation, and application of these algorithms in CD and UC.

## Conclusions

This study showed that 60% or more of patients with CD or UC had an inadequate response to their first advanced therapies within 1 year after initiation. The inadequate responses were mostly driven by low adherence and switching to or addition of a new treatment. Health plan claims data appear useful to classify inadequate responders in CD or UC and additional research should be done to further validate this claims-based algorithm in a clinical setting.

## Supplementary Information

**Additional file 1. Table S1.** Details of the algorithm used to identify inadequate response for Crohn’s disease and ulcerative colitis. **Table S2.** Current procedural terminology codes for Crohn’s disease and ulcerative colitis surgery. **Table S3.** Oral corticosteroid conversions table [22–24]. **Figure S1.** Patient identification.

## Data Availability

The HealthCore database is a proprietary health claims database and is not accessible to the public. HealthCore researchers were provided access pursuant to Health Insurance Portability and Accountability Act. Further information concerning access to HealthCore’s database may be provided upon request; please contact Michael Grabner at mgrabner@healthcore.com.

## References

[1] Alatab S, Sepanlou SG, Ikuta K, Vahedi H, Bisignano C, Safiri S, Sadeghi A, Nixon MR, Abdoli A, Abolhassani H, Alipour V (2020). The global, regional, and national burden of inflammatory bowel disease in 195 countries and territories, 1990–2017: a systematic analysis for the Global Burden of Disease Study 2017. Lancet Gastroenterol Hepatol..

[2] Dahlhamer JM, Zammitti EP, Ward BW, Wheaton AG, Croft JB (2016). Prevalence of inflammatory bowel disease among adults aged ≥18 years—United States, 2015. MMWR Morb Mortal Wkly Rep.

[3] Crohn’s and Colitis Foundation of America. The facts about inflammatory bowel disease. 2021. Available from: http://www.crohnscolitisfoundation.org/assets/pdfs/updatedibdfactbook.pdf.

[4] Fact Sheet. News from the IBD Help Center. Janus kinase inhibitors (JAK inhibitors). Crohn’s and Colitis Foundation 2018 [Available from: https://www.crohnscolitisfoundation.org/sites/default/files/legacy/assets/pdfs/jak-inhibitors.pdf.

[5] Lichtenstein GR, Loftus EV, Isaacs KL, Regueiro MD, Gerson LB, Sands BE (2018). ACG clinical guideline: management of Crohn’s disease in adults. Am J Gastroenterol.

[6] Rubin DT, Ananthakrishnan AN, Siegel CA, Sauer BG, Long MD (2019). ACG clinical guideline: ulcerative colitis in adults. Am J Gastroenterol.

[7] Ben-Horin S, Kopylov U, Chowers Y (2014). Optimizing anti-TNF treatments in inflammatory bowel disease. Autoimmun Rev.

[8] Papamichael K, Cheifetz AS (2019). Therapeutic drug monitoring in inflammatory bowel disease: For every patient and every drug?. Curr Opin Gastroenterol.

[9] Perera S, Yang S, Stott-Miller M, Brady J (2018). Analysis of healthcare resource utilization and costs after the initiation of biologic treatment in patients with ulcerative colitis and Crohn’s disease. J Health Econ Outcomes Res.

[10] Brady JE, Stott-Miller M, Mu G, Perera S (2018). Treatment patterns and sequencing in patients with inflammatory bowel disease. Clin Ther.

[11] Armuzzi A, DiBonaventura M, Tarallo M, Lucas J, Bluff D, Hoskin B (2020). Treatment patterns among patients with moderate-to-severe ulcerative colitis in the United States and Europe. PLoS ONE.

[12] Chen G, Lissoos T, Dieyi C, Null KD (2021). Development and validation of an inflammatory bowel disease severity index using US administrative claims data: a retrospective cohort study. Inflamm Bowel Dis.

[13] Ye Y, Manne S, Bennett D (2019). Identifying patients with inflammatory bowel diseases in an administrative health claims database: Do algorithms generate similar findings?. Inquiry.

[14] Osterman MT, Haynes K, Delzell E, Zhang J, Bewtra M, Brensinger C (2014). Comparative effectiveness of infliximab and adalimumab for Crohn’s disease. Clin Gastroenterol Hepatol.

[15] Osterman MT, Haynes K, Delzell E, Zhang J, Bewtra M, Brensinger CM (2015). Effectiveness and safety of immunomodulators with anti-tumor necrosis factor therapy in Crohn’s disease. Clin Gastroenterol Hepatol.

[16] Curtis JR, Baddley JW, Yang S, Patkar N, Chen L, Delzell E (2011). Derivation and preliminary validation of an administrative claims-based algorithm for the effectiveness of medications for rheumatoid arthritis. Arthritis Res Ther.

[17] Hunter T, Grabner M, Birt J, Isenberg K, Shan M, Teng CC (2022). Identifying inadequate response among patients with ankylosing spondylitis and psoriatic arthritis prescribed advanced therapy in a real-world, commercially insured adult population in the USA. Clin Rheumatol.

[18] Chan W, Chen A, Tiao D, Selinger C, Leong R (2017). Medication adherence in inflammatory bowel disease. Intest Res.

[19] Null KD, Xu Y, Pasquale MK, Su C, Marren A, Harnett J (2017). Ulcerative colitis treatment patterns and cost of care. Value Health.

[20] Ruffolo C, Scarpa M, Bassi N (2010). Infliximab, azathioprine, or combination therapy for Crohn’s disease. N Engl J Med.

[21] Bokemeyer B, Picker N, Wilke T, Rosin L, Patel H (2022). Inadequate response, treatment patterns, health care utilization, and associated costs in patients with ulcerative colitis: retrospective cohort study based on German claims data. Inflamm Bowel Dis.

[22] Czock D, Keller F, Rasche FM, Häussler U (2005). Pharmacokinetics and pharmacodynamics of systemically administered glucocorticoids. Clin Pharmacokinet..

[23] Markham A, Bryson HM (1995). A review of its pharmacological properties and therapeutic efficacy. Drugs..

[24] Meikle AW, Tyler FH (1977). Potency and duration of action of glucocorticoids. Effects of hydrocortisone, prednisone and dexamethasone on human pituitary-adrenal function. Am J Med..

