# Supplementary material for: Identification of inadequate responders to advanced therapy among commercially-insured adult patients with Crohn’s disease and ulcerative colitis in the United States
Source: BMC Gastroenterol. 2023 Mar 9;23:63. doi: 10.1186/s12876-023-02675-w (PMC9996947; doi:10.1186/s12876-023-02675-w)
Supplement: Supplementary file 1 — Additional file 1. Table S1. Details of the algorithm used to identify inadequate response for Crohn’s disease and ulcerative colitis. Table S2. Current procedural terminology codes for Crohn’s disease and ulcerative colitis surgery. Table S3. Oral corticosteroid conversions table [22–24]. Figure S1. Patient identification. [file 12876_2023_2675_MOESM1_ESM.docx]

**SUPPLEMENT**

**Table S1. Details of the Algorithm Used to Identify Inadequate Response for Crohn’s Disease and Ulcerative Colitis**

| **Criteria** | **Description and implementation** |
| --- | --- |
| Low adherence to index medication  Low adherence = 1 | Defined as Proportion of Days Covered (PDC) < 80%. PDC was calculated as the ratio of the total number of days with drug at hand to the length of the time period under investigation (365 days).  Total number of days with drug at hand was calculated as follows:   1. For outpatient Rx claims, the days’ supply values were summed across all claims for the index drug. 2. For medical claims, days’ supply was equal to the labelled maintenance frequency:    1. Adalimumab: 14 days (SC)    2. Certolizumab: 28 days (SC)    3. Golimumab: 56days (IV), 28 days (SC)    4. Infliximab: 56 days    5. Tofacitinib: 1 day (Tab)    6. Ustekinumab, Stelara infusion only for Crohn’s Disease patients: 56 days    7. Natalizumab: 28 days (SC)    8. Vedolizumab: 56 days (IV)   In both cases, overlapping days’ supply across fills were subtracted from the total (with total Rx overlapping days across all fills being capped at 14 days; no cap for medical claims). If multiple claims occur on the same date, further cleaning may be applied. |
| Switch/add non-index advanced therapies  Advanced therapy switch/add = 1 | Patients who initiate other non-index advanced therapy medications during post-index period (365 days) were flagged. Only on-label switch/addition was used for purposes of the algorithm. |
| Add of new conventional therapy  Conventional therapy add = 1 | Indicator for patient who initiated therapy during 12 months post-index period with a new conventional therapy that they were not already taking during the pre-index period |
| Increase in dose or frequency of index advanced therapy  Dose increase = 1 | Dose escalation was assessed by comparing the first observed dose for the index advanced therapy after loading to all doses observed during the remainder of follow-up. If any dose was at least 20% higher than the index dose, the patient was flagged as having escalated their dose. Only the index advanced therapy was assessed.  Dose will be calculated as follows:   - - 1. For outpatient Rx claims:   Dose = (Strength x Quantity Dispensed (QD))/Days’ supply   - - 1. For medical claims:   Dose = (Strength based on HCPCS code) x (Billed units)  (The total dosing amount was used without considering the time window between two administrations.)  For patients who only received IV administrations, the number of claims was calculated. This frequency must be within 120% of the number expected during 12 months time period based on guidelines. The following number of infusions by drug was considered as a frequency increase and flagged:   1. Golimumab: ≥ 10 claims 2. Infliximab: ≥ 12 claims 3. Natalizumab: ≥ 16 claims 4. Vedolizumab: ≥ 10 claims   For ustekinumab, criterion was not applicable as only a single IV administration is used. |
| Increase in dose of oral glucocorticoid  Increase = 1 | For patients who received no prescriptions for oral glucocorticoids during the 6 months prior to the index date, cannot receive more than 30 days of oral glucocorticoids (as measured by days’ supply; equal to >1 fill) between index date + 90 days and index date + 365 days, inclusive.  For patients who received prescriptions for oral glucocorticoids in the 6 months prior to the index date, the cumulative glucocorticoid dose in the 6 months prior to end of follow-up (index date+184 to index date+365) must be similar (that is, within 120%) to the cumulative dose in the 6 months prior to the index visit date.  Cumulative dose was calculated as the sum, across all fills, of [Strength x QD x Corticosteroid equivalent dose (see Table S3)] on each fill; only applied to oral glucocorticoids.  Both medical and pharmacy claims were assessed. |
| Use of new pain medication class not observed at pre-index  New treatment class = 1 | Indicator for patients who initiated therapy during 12 months post-index period with a new pain medication class that they were not already taking during pre-index period.  Classes included opioids, NSAIDs, non-narcotic analgesics, neuromodulators. Use of a new drug at follow-up that was in the same class as drugs used at pre-index were not flagged. Both medical and pharmacy claims were assessed. |
| Surgery for UC/CD  Treatment = 1 | Indicator for claims for the listed surgery procedures in the applicable disease area at any time during the 12 months post-index period. This was assessed in medical claims only. |

Table S2. Current Procedural Terminology Codes for Crohn’s Disease and Ulcerative Colitis Surgery

| **Current Procedural Terminology codes** | **Description** |
| --- | --- |
| 44160 | Colectomy, partial, with removal of terminal ileum with ileocolostomy |
| 44205 | Laparoscopy, surgical; colectomy, partial, with removal of terminal ileum with ileocolostomy |
| 44120 | Enterectomy, resection of small intestine; single resection and anastomosis |
| 46270 | Surgical treatment of anal fistula (fistulectomy/fistulotomy); subcutaneous |
| 46050 | Incision and drainage, perianal abscess, superficial |
| 44310 | Ileostomy or jejunostomy, non-tube |
| 46060 | Incision and drainage of ischiorectal or intramural abscess, with fistulectomy or fistulotomy, submuscular, with or without placement of seton |
| 44140 | Colectomy, partial; with anastomosis |
| 44187 | Laparoscopy, surgical; ileostomy or jejunostomy, non-tube |
| 44202 | Laparoscopy, surgical; enterectomy, resection of small intestine, single resection and anastomosis |
| 46280 | Surgical treatment of anal fistula (fistulectomy/fistulotomy); transsphincteric, suprasphincteric, extrasphincteric or multiple, including placement of seton, when performed |
| 44204 | Laparoscopy, surgical; colectomy, partial, with anastomosis |
| 44150 | Colectomy, total, abdominal, without proctectomy; with ileostomy or ileoproctostomy |
| 44155 | Colectomy, total, abdominal, with proctectomy; with ileostomy |
| 44158 | Colectomy, total, abdominal, with proctectomy; with ileoanal anastomosis, creation of ileal reservoir (S or J), includes loop ileostomy, and rectal mucosectomy, when performed |
| 44210 | Laparoscopy, surgical; colectomy, total, abdominal, without proctectomy, with ileostomy or ileoproctostomy |
| 44211 | Laparoscopy, surgical; colectomy, total, abdominal, with proctectomy, with ileoanal anastomosis, creation of ileal reservoir (S or J), with loop ileostomy, includes rectal mucosectomy, when performed |
| 44212 | Laparoscopy, surgical; colectomy, total, abdominal, with proctectomy, with ileostomy |
| 45113 | Proctectomy, partial, with rectal mucosectomy, ileoanal anastomosis, creation of ileal reservoir (S or J), with or without loop ileostomy |
| 45119 | Proctectomy, combined abdominoperineal pull-through procedure (eg, colo-anal anastomosis), with creation of colonic reservoir (eg, j-pouch), with diverting enterostomy when performed |
| 45397 | Laparoscopy, surgical; proctectomy, combined abdominoperineal pull-through procedure (eg, colo-anal anastomosis), with creation of colonic reservoir (eg, J-pouch), with diverting enterostomy, when performed |

Table S3. Oral corticosteroid conversions table (22-24)

| **Compound** | **Equivalent Dose (mg)** |
| --- | --- |
| Betamethasone | 0.75 |
| Budesonide | 0.375 |
| Cortisone | 25 |
| Deflazacort | 7.5 |
| Dexamethasone (Decadron) | 0.75 |
| Hydrocortisone | 20 |
| Methylprednisolone (12-36 Hours) | 4 |
| Paramethasone | 2 |
| Prednisolone | 5 |
| Prednisone | 5 |
| Triamcinolone | 4 |

**Figure S1. Patient identification**

**
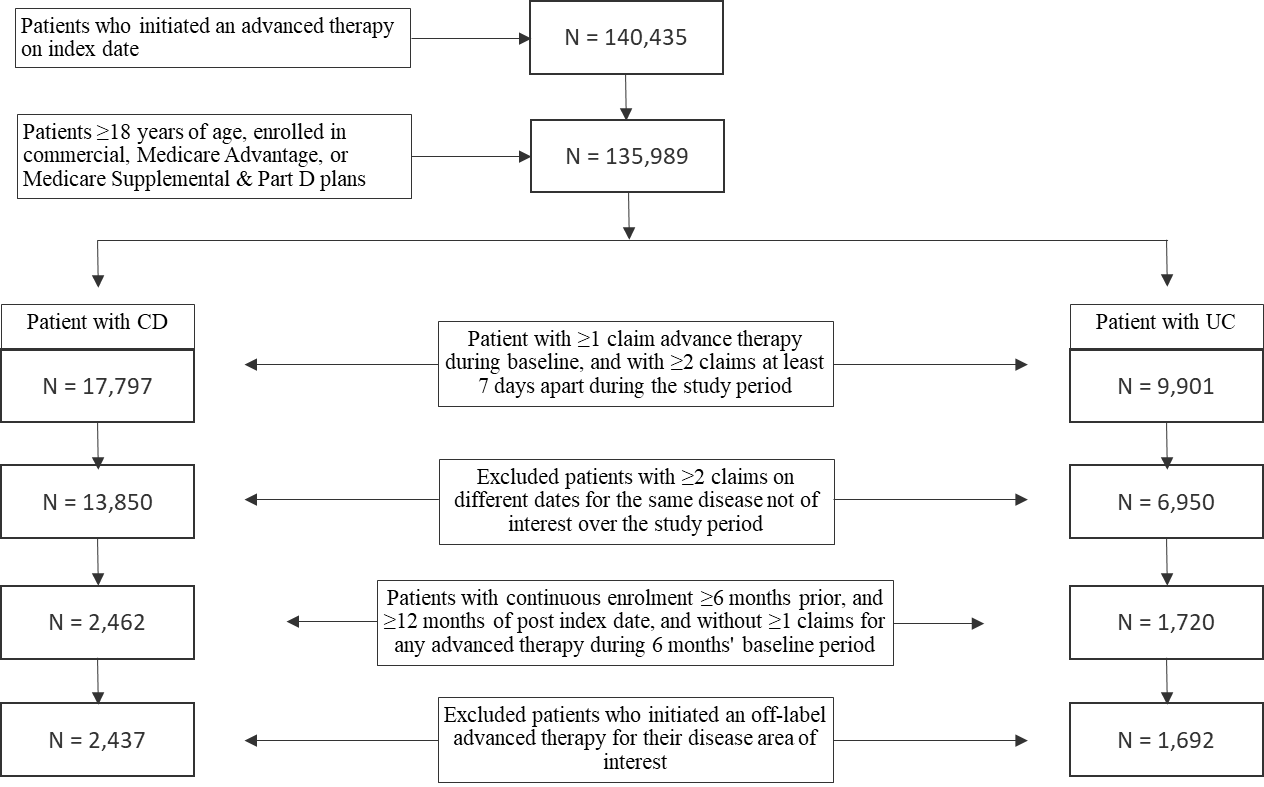
**
